# Applications of bone regenerative medicine in the foot and ankle: mechanisms, technologies, and therapeutic advances

**DOI:** 10.3389/fbioe.2025.1653964

**Published:** 2025-12-02

**Authors:** Lianbo Yang, Yijie Li, Tianqi Wang, Zhuo Zhao, Wang Li, Jinghang Lv, Haoran Chen, Zhaodong Qi, Xinming Wang, Wenchi Bao, Haidong Liang

**Affiliations:** 1 Regenerative Medicine and Wound Repair Center, The Second Affiliated Hospital of Dalian Medical University, Dalian, China; 2 Dalian Medical University, Dalian, China; 3 Department of Joint Surgery, People’s Hospital of Liaoning Province, Shenyang, China; 4 Shengjing Hospital of China Medical University Department of Radiology, Shenyang, China

**Keywords:** regenerative medicine, foot and ankle diseases, platelet-rich plasma, stem cells, 3D bioprinting

## Abstract

**Background:**

Foot and ankle diseases significantly impact quality of life, with regenerative medicine emerging as a promising approach. A comprehensive evaluation of both efficacy and safety is paramount for its clinical translation.

**Methods:**

A comprehensive literature search was conducted in PubMed using keywords “regenerative medicine” and “foot and ankle” (as of 31 December 2024). Studies were categorized by technology and disease.

**Results:**

PRP and HA showed short-term efficacy in talar cartilage repair; stem cells enhanced functional recovery in ankle osteoarthritis. 3D printing enabled personalized implants. Exosomes and AI were identified as future directions. However, the reporting of safety data was often sporadic and non-standardized, highlighting the need for more systematic monitoring in future studies.

**Conclusion:**

Regenerative therapies demonstrate potential but require further validation through robust trials that prioritize standardized safety reporting alongside efficacy outcomes. Gaps in exosome isolation, long-term safety, and clinical translation need addressing.

## 1 Introduction

Foot and ankle disorders, stemming from their complex anatomy and weight-bearing function (Riddick et al., 2019; Brockett and Chapman, 2016), represent a significant clinical challenge worldwide (Lin et al., 2006). Current foot and ankle treatments include surgical and conservative options, with the latter often offering lower risk and cost alongside better structural preservation (Donken et al., 2012; Kerkhoffs et al., 2007; Ramelli et al., 2024; Lan et al., 2021a). Therefore, exploring more effective treatment methods, such as regenerative medicine, is crucial for improving patient outcomes and quality of life.

Regenerative medicine focuses on structural and functional restoration primarily through pharmacological activation of endogenous repair, cell-based replacement therapies, and bioengineered tissue constructs (Bajaj et al., 2014). This review performs a scoping analysis of several key approaches (e.g., stem cells (Golchin, 2022), PRP (Everts et al., 2020),3D printing, (Tack et al., 2016),and exosomes (Lai et al., 2022)) and their translational applications in foot and ankle pathology.

Despite rapid advancements, the evidence for these regenerative applications remains fragmented. A comprehensive synthesis that maps the current landscape, evaluates the efficacy and safety of different technologies across specific pathologies, and identifies key future directions is required. Consequently, this scoping review aims to critically explore the applications of bone regenerative medicine in the foot and ankle. We systematically categorize and evaluate the evidence for key technologies—including stem cells, platelet-rich plasma, hyaluronic acid, hypertonic glucose, placental tissues, 3D printing, exosomes, and AI—in managing major conditions such as talar osteochondral lesions, ankle osteoarthritis, Achilles tendon injuries, plantar fasciitis, and ligament injuries. This review seeks to provide a clear overview of the state of the art, discuss translational challenges, and inform future clinical research and practice.

## 2 Materials

This scoping review was conducted in accordance with the Preferred Reporting Items for Systematic Reviews and Meta-Analyses extension for Scoping Reviews (PRISMA-ScR) guidelines and followed the methodological framework proposed by Arksey and O’Malley. The protocol was registered in advance. The methodology comprised five key stages: (1) identifying the research questions, (2) identifying relevant studies, (3) study selection, (4) charting the data, and (5) collating, summarizing, and reporting the results.

Step 1: Identifying the Research Questions

The primary objectives of this scoping review were as follows:

1. To map the key regenerative medicine technologies (e.g., stem cells, platelet-rich plasma, 3D printing) used in the treatment of common foot and ankle diseases.
2. To examine and describe the application and scope of these technologies in specific foot and ankle disorders, including talar cartilage injuries, ankle osteoarthritis, Achilles tendon injuries, plantar fasciitis, and ligament injuries.
3. To synthesize and report the main research outcomes and findings of various regenerative therapies in clinical applications.
4. To identify emerging technological trends (e.g., exosomes, artificial intelligence) and research gaps in the current literature, and to suggest directions for future research.

Step 2: Identifying Relevant Studies

A comprehensive literature search was performed in PubMed from inception to 31 December 2024. The search strategy combined Medical Subject Headings (MeSH) and free-text terms related to regenerative medicine and foot and ankle pathologies. The search terms included but were not limited to: “regenerative medicine,” “stem cells,” “platelet-rich plasma,” “PRP,” “3D printing,” “biomaterials,” “exosomes,” “growth factors,” “talus cartilage injuries,” “ankle osteoarthritis,” “Achilles tendon injury,” “plantar fasciitis,” and “ligament injury.” The full search strategy for PubMed is illustrated in Table 1. The search was limited to English-language publications. Additionally, the reference lists of included studies were manually screened to identify any potentially relevant articles.

**TABLE 1:** Search strategy for PubMed.

#1	Regenerative medicine [Mesh]
#2	Regenerative therap[Title/Abstract] OR tissue engineering[Title/Abstract] OR stem cell[Title/Abstract] OR platelet-rich plasma[Title/Abstract] OR PRP[Title/Abstract] OR biomaterial[Title/Abstract] OR 3D print[Title/Abstract] OR exosome[Title/Abstract] OR “growth factor”[Title/Abstract]
#3	#1 OR #2
#4	Talus [Mesh] OR Ankle Joint [Mesh] OR Achilles Tendon [Mesh] OR Plantar Fasciitis [Mesh] OR Ankle Injuries [Mesh] OR Ligaments, Articular [Mesh]
#5	Talus Cartilage Injury[Title/Abstract] OR osteochondral lesion of talus[Title/Abstract] OR OLT[Title/Abstract] OR ankle osteoarthr[Title/Abstract] OR ankle OA[Title/Abstract] OR Achilles tendinopath[Title/Abstract] OR Achilles rupture[Title/Abstract] OR plantar fasciitis[Title/Abstract] OR heel spur[Title/Abstract] OR ankle sprain[Title/Abstract] OR ligament injury[Title/Abstract] OR lateral ankle instability[Title/Abstract] OR “foot and ankle”[Title/Abstract]
#6	#4 OR #5
#7	#3 AND #6

The PICOS framework (Population, Intervention, Comparison, Outcome, Study Design) was applied to define eligibility criteria, as detailed in Table 2.

**TABLE 2:** PICOS framework for inclusion criteria.

Category	Criteria
Population	Patients diagnosed with foot and ankle disorders (e.g., talar cartilage injury, ankle osteoarthritis, Achilles tendon injury, plantar fasciitis, ligament injury) or corresponding animal models.
Intervention	Treatment involving at least one regenerative therapy (e.g., stem cells, PRP, hyaluronic acid, 3D-printed scaffolds, exosomes).
Comparison	Any comparator (e.g., placebo, conventional treatment, no treatment).
Outcomes	Clinical, functional, radiological, or histological outcomes related to foot and ankle diseases (e.g., VAS pain score, AOFAS score, tendon healing, cartilage repair).
Study Design	Clinical studies (RCTs, non-RCTs, cohort studies, case series) and preclinical basic studies.

Studies were excluded if they were: (1) non-English publications; (2) non-peer-reviewed articles (e.g., editorials, commentaries, conference abstracts); (3) protocols or studies with unavailable full text; or (4) irrelevant to regenerative medicine or foot and ankle diseases.

Step 3: Study Selection

The study selection process involved two phases. First, two reviewers independently screened titles and abstracts against the eligibility criteria. Second, the full texts of potentially eligible studies were retrieved and assessed independently by the same reviewers. Any disagreements were resolved through discussion or by a third reviewer. The study selection process is summarized in a PRISMA flow diagram, which outlines the number of records identified, included, and excluded at each stage.

Step 4: Charting the Data

A standardized data extraction form was developed and piloted to document key information from included studies. Data were extracted by one reviewer and verified by another. The extracted items included: first author, publication year, study design, sample size, patient/model characteristics, intervention details (type, preparation, dosage, etc.), comparator, follow-up duration, and main outcomes/findings, as shown in Table 3.

**TABLE 3:** Preliminary standardized data extraction items.

Category	Specific data items for extraction
1. Publication content	First Author Publication Year Article Title Journal/Source Study Type (e.g., RCT, Cohort, Case Series, Preclinical Study) Funding Source
2. Participant characteristics	Population/Model (e.g., Patients with OLT, Rat model of ankle OA) Sample Size (n) Mean Age Sex Ratio (Male/Female) Disease Severity or Classification (e.g., Lesion size, Kellgren-Lawrence grade)
3. Intervention details	Type of Regenerative Technology(e.g., PRP, BMAC, ADSCs, HA, Hypertonic Glucose, Placental Tissue, 3D-printed scaffold, Exosomes) Specific Preparation Protocol(e.g., PRP centrifugation method, Cell source and dosage, Scaffold material) Intervention Protocol(Dose, Concentration, Number of injections, Frequency, Follow-up time) Combination Therapies(e.g., Used alongside microfracture, debridement, etc.)
4. Comparison details	Type of Comparison (e.g., Placebo, No treatment, Conventional therapy, Other active treatment) Specific Details of the Comparator Intervention
5. Outcome measures	Primary Outcomes: Pain scores (e.g., VAS), Functional scores (e.g., AOFAS, VISA-A), Imaging scores (e.g., MOCART) Secondary Outcomes: Histological results, Return-to-sport time, Adverse events, Patient satisfaction Key Findings and Author’s Conclusions
6. Methodology and limitations	Main Study Limitations as reported by authors Authors’ Recommendations for Future Research

Step 5: Collating, Summarizing, and Reporting Results

The extracted data were summarized quantitatively (e.g., frequency analysis) and qualitatively (narrative synthesis). The analysis aimed to describe the characteristics, scope, and trends of regenerative therapies in foot and ankle disorders. Results were presented in tables and narrative form.

Consistent with the purpose of a scoping review, no formal quality assessment of included studies was conducted, as the goal was to map the evidence rather than evaluate intervention efficacy.

## 3 Results

**FIGURE 1:**
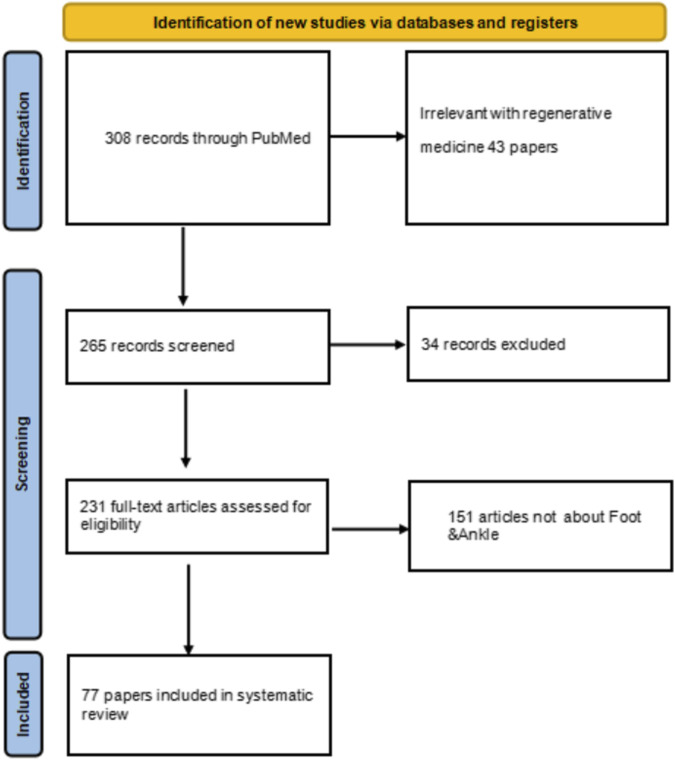
Literature search diagram.

**FIGURE 2:**
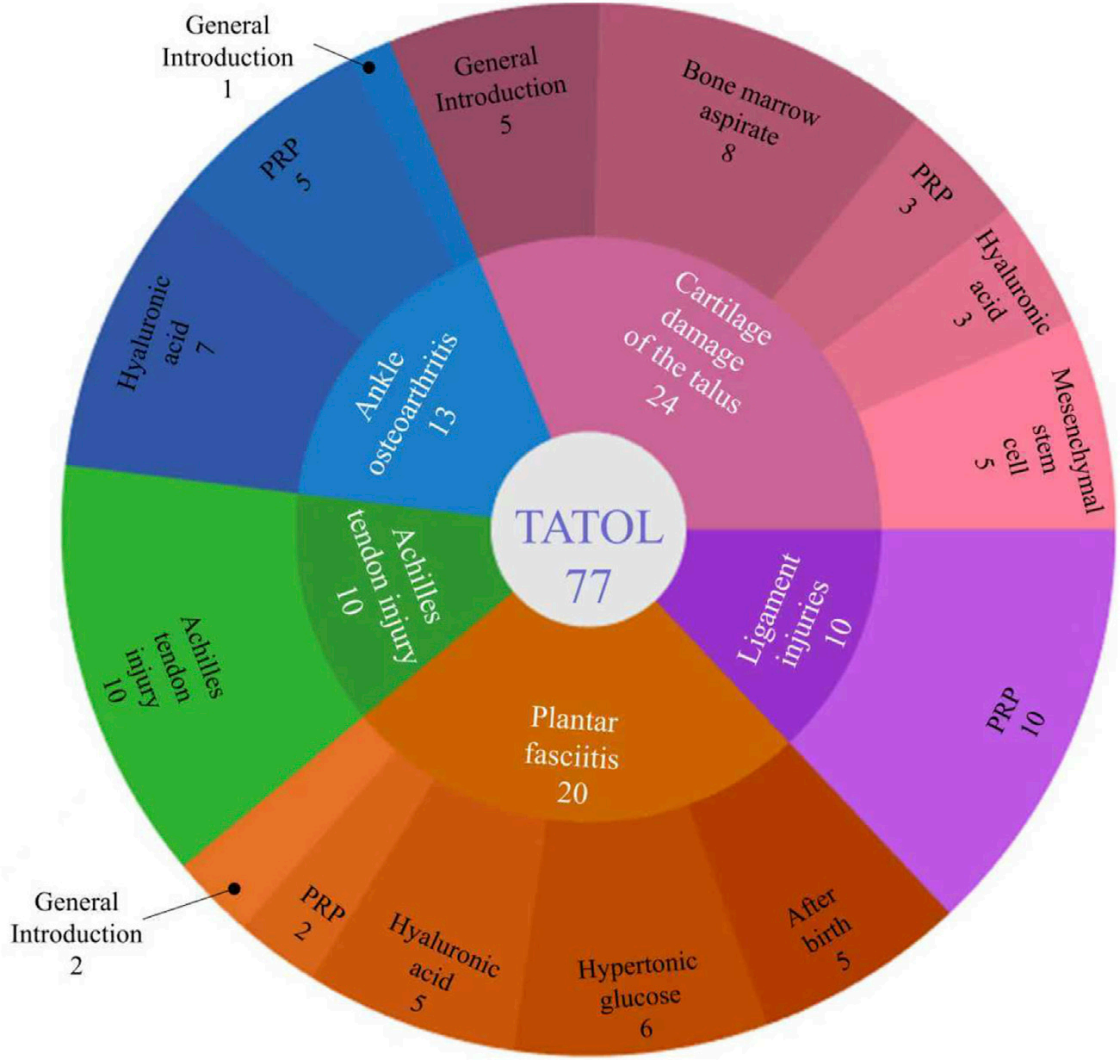
Number of references in each section of this paper regarding the application of regenerative medicine to diseases.

A systematic search of the PubMed database yielded 308 records for initial screening. Following the removal of duplicates, 265 records remained for title and abstract screening. During this stage, 154 records were excluded as they were deemed irrelevant to the focus on foot and ankle diseases. Subsequently, 111 full-text articles were assessed for eligibility. Of these, 43 articles were excluded for not meeting the regenerative medicine intervention criteria, and 34 articles were excluded due to unavailability of full text or being non-peer-reviewed publications. Ultimately, a total of 77 studies were included in the qualitative synthesis and descriptive analysis presented in this scoping review (see flow diagram in Figure 1). The included studies were further categorized and mapped based on the specific regenerative technology and foot/ankle disorder investigated (see study classification in Figure 2).

### 3.1 Applications in disease management

The standardized scoring tools commonly used for postoperative assessment of foot and ankle diseases and their clinical characteristics are summarized in Table 4. These tools cover multiple dimensions of evaluation, including pain, function, and imaging. Among them, the MOCART score provides a quantitative basis for evaluating cartilage repair efficacy through objective imaging parameters, while the VISA-A and Tegner scores focus on longitudinal monitoring of functional recovery in sports activities.

**TABLE 4:** Scoring tools for postoperative assessment of foot and ankle diseases.

Scoring tool	Score range	Description	References
AOFAS (American Orthopedic Foot and Ankle Society) Score	0–100	Assesses foot and ankle function, pain, and alignment, commonly used for conditions like plantar fasciitis.	Vosoughi et al. (2018)
FHSQ (Foot Health Status Questionnaire)	Variable	Assesses foot health and quality of life, including pain, function, and daily activities.	Riskowski et al. (2011)
VAS (Visual Analog Scale)	0–10	A subjective pain assessment tool where patients mark their pain intensity.	Hawker et al. (2011)
MOCART (Magnetic Resonance Observation of Cartilage Repair Tissue) Score	Variable	Uses MRI to evaluate tissue quality and recovery after cartilage repair.	Schreiner et al. (2021)
VISA-A (Victorian Institute of Sports Assessment - Achilles) Score	0–100	Assesses function and symptom severity in patients with Achilles tendinopathy.	Gatz et al. (2020)
EQ-VAS (EuroQol Visual Analog Scale)	0–100	Assesses overall health status, where patients rate their current health experience.	Lin et al. (2023)
Tegner Score	0–10	Assesses activity level and functional status, suitable for knee and ankle joint rehabilitation.	Briggs et al. (2009)
FAAM (Foot and Ankle Ability Measure)	0–100	Assesses foot and ankle function and quality of life, particularly in chronic conditions.	Martin et al. (2005)
AHFS (Ankle-Hindfoot Scale)	0–100	Evaluates ankle joint function recovery, commonly used in postoperative rehabilitation.	Nijmeijer et al. (2023)
FADI (Foot and Ankle Disability Index)	0–100	Assesses the degree of foot and ankle disability, suitable for various foot and ankle conditions.	Omeragic et al. (2023)
MOXFQ (Manchester-Oxford Foot Questionnaire)	0–100	Assesses the impact of foot diseases on quality of life, used before and after foot surgery.	Dawson et al. (2011)

#### 3.1.1 Talus cartilage injuries

Osteochondral lesions of the talus (OLT) refer to localized damage to the articular cartilage and its underlying subchondral bone, including cartilage tears, subchondral bone fractures, bone marrow edema, and subchondral cysts. These injuries are of significant concern in ankle joint pathology, commonly occurring following sprains, dislocations, or fractures (Cheng and Wang, 2024). Studies report that approximately 40% of patients with ankle sprains experience long-term instability and recurrent sprains, which can further lead to cartilage damage. (Wang et al., 2020). Treatment largely depends on clinical symptoms, the location of the lesions, and imaging findings. For lesions located medially or laterally with no radiological evidence of cartilage fragment detachment, conservative treatment may be considered. Conservative options include rest, casting or bracing, weight-bearing restrictions, and the use of nonsteroidal anti-inflammatory drugs. Surgical treatments encompass arthroscopic debridement, bone marrow stimulation or microfracture, osteochondral autograft transplantation, autologous chondrocyte implantation, and allograft osteochondral transplantation, among other techniques (Bruns et al., 2021). Among these, microfracture has traditionally been considered the “gold standard” for initial treatment as it promotes the formation of fibrocartilage at cartilage defects (Nguyen et al., 2024). According to the German Society for Orthopedics and Trauma Surgery, BMS is recommended for lesions with an area smaller than 1.5 cm^2^ and a depth less than 5 mm (Walther et al., 2024).

##### 3.1.1.1 PRP

Intra-articular injection of PRP has been used in the treatment of talus cartilage lesions and has shown a significant reduction in pain scores, with functional improvement lasting for at least 6 months (Mei-Dan et al., 2012). When combined with arthroscopic microfracture surgery, PRP further enhances functional scores for mid-stage osteochondral lesions (Guney et al., 2015). Moreover, PRP is increasingly considered a primary adjunctive treatment after OCL surgery (Gormeli et al., 2015).

##### 3.1.1.2 HA

Hyaluronic acid (HA), a nonsulfated linear polysaccharide comprising repeating disaccharides, exhibits tissue-specific molecular weight variations and high hydration *in vivo* (Prestwich, 2011). Its physicochemical properties are tunable via crosslinking/degradation, facilitating angiogenesis, osteointegration, and cellular homeostasis (Allison and Grande-Allen, 2006). Intra-articular injection of HA is another effective treatment for talus cartilage lesions, significantly reducing pain scores and improving functional status for at least 6 months (Mei-Dan et al., 2012). HA has also shown superior healing effects when used as an adjunct to arthroscopic microfracture surgery for osteochondral lesions (Shang et al., 2016), contributing to better outcomes in cartilage healing (Doral et al., 2012), and joint function recovery.

##### 3.1.1.3 BMAC

Bone marrow aspirate concentrate (BMAC) transplantationhas proven to be an effective regenerative technique for talus cartilage defects (Buda et al., 2015; Desando et al., 2017), with clinical outcomes slightly better than those of autologous chondrocyte implantation (Buda et al., 2015). BMAC, when used in combination with microfracture surgery, significantly reduces the recurrence rate of osteochondral lesions (Murphy et al., 2019). BMAC combined with arthroscopic microfracture surgery provides better functional improvement in the medium term. When combined with HA and fibrin, BMAC significantly enhances ankle joint cartilage function (Abas et al., 2022). However, not all combination therapies yield favorable clinical results, as seen in studies combining extracellular matrix allografts with cBMA (Mercer et al., 2022) or cBMA with autologous bone tissue grafts, which showed no significant benefits (Shimozono et al., 2019).

##### 3.1.1.4 Adipose-derived MSCs and stromal vascular fraction

Adipose-derived mesenchymal stem cells have shown promising results in treating talus cartilage lesions and have been shown to be safe in the treatment of ankle osteoarthritis pain (Natali et al., 2021). In patients with ankle osteoarthritis undergoing subtalar medial oblique osteotomy or calcaneal sliding osteotomy, additional mesenchymal stem cells (MSCs) injection combined with bone marrow stimulation significantly improved VAS and AOFAS scores in the short-term follow-up (Kim et al., 2016; Kim and Koh, 2016). In combination therapies, MSCs have been widely applied. Kim et al. (2014a) compared the effects of bone marrow stimulation alone with bone marrow stimulation combined with MSC-containing stromal vascular fraction injections. The results indicated that the combined therapy of MSC-SVF and bone marrow stimulation significantly improved VAS, AOFAS, Tegner, and MOCART scores compared to bone marrow stimulation alone. Kim et al. (2013) found that combined treatment with MSCs had better results in the treatment of OLT in patients over 50 years of age by comparing MSC injection plus arthroscopic bone marrow stimulation versus arthroscopic bone marrow stimulation alone for the treatment of OLT in elderly patients.

The main information of Talus Cartilage Injuries included literature is shown in Table 5.

**TABLE 5:** Summary of papers on talus cartilage injuries.

References	Sample size	Method	Scoring system	Results	Conclusion
Mei-Dan et al. (2012)	30	15 PRP, 15 HA, 3intra-articular injections for 28 weeks	AOFAS, AHFS, VAS	VAS: Significant decrease (P < 0.001), lowest at 12 weeks (P < 0.001), slight increase at 24 weeks	Intra-articular PRP and HA significantly reduced pain and improved function for at least 6 months.
Guney et al. (2015)	35	16 control, 19 PRP + microfracture, 16.2 months follow-up	AOFAS, FAAM, VAS	AOFAS: 89.2 ± 3.9 (PRP) vs. 71.0 ± 10.2 (control)	PRP as an adjunct to microfracture improved functional scores in the mid-term.
Gormeli et al. (2015)	40	13 PRP, 14 HA, 13 saline, 15.3 months follow-up	AOFAS, VAS	AOFAS: PRP group significantly higher than HA and control, VAS significantly lower	PRP should be considered as the primary adjunctive therapy for osteochondral lesion surgery.
Shang et al. (2016)	35	17 control, 18 HA injections	MRI, AOFAS, VAS	MRI: Injection group better thickness and T2 index (P < 0.01), higher improvement in AOFAS and VAS (P < 0.05).	Intra-articular HA injections may provide better functional recovery when combined with microfracture.
Doral et al. (2012)	57	16 control, 41 HA injections	Freiburg, AOFAS	Injection group significantly improved compared to non-injection group	Intra-articular HA injections are effective as an adjunct to microfracture for osteochondral lesions.
Buda et al. (2015)	80	40 ACI, 40BMDCT	AOFAS, MRI MOCART, T2	AOFAS: BMDCT 94.7, ACI 93.9, both groups similar in MOCART and T2	Both ACI and BMDCT are effective for treating osteochondral lesions, with BMDCT showing potentially superior outcomes.
Desando et al. (2017)	22	7 mACI, 15 mBMAC	AOFAS, histologic and immunohistochemical assessments	AOFAS: mACI 92.4, mBMAC 84.22 at 36 months (P < 0.05), mACI superior	mACI and mBMAC both showed effectiveness, with mACI yielding superior results.
Murphy et al. (2019)	101	52 microfracture alone, 49 microfracture + BMAC, 36-month follow-up		Revision rate: Microfracture 28.8%, microfracture + BMAC 12.2%	Microfracture combined with BMAC reduces revision rates for osteochondral lesions.
Hannon et al. (2016)	34	34BMS, 22BMAC + BMS	FAOS, SF-12	Significant improvement in BMAC group (P < 0.01), higher T2 relaxation in BMAC/BMS group (P = 0.030 and P < 0.001)	BMAC combined with bone marrow stimulation results in better functional outcomes.
Abas et al. (2022)	94	BMC + HA + fibrin	MOXFQ	Significant improvement at 12 months	BMC combined with HA and fibrin showed good safety and tolerance.
Mercer et al. (2022)	60	26 AOT + EMCA/BMAC, 34 AOT/CBMA	MRI MOCART	Both groups significantly improved, no difference between AOT + EMCA and AOT + CBMA	Both AOT + EMCA and AOT + CBMA showed significant improvement, with no notable difference between treatments.
Shimozono et al. (2019)	54	28 AOT + CBMA, 26 AOT	FAOS, SF-12	FAOS:AOT + CBMA: Pre-op 52.3, Post-op 75.5; AOT 40.7, Post-op 69.6	AOT treatment is effective, combined with CBMA treatment effect enhancement is not significant
Natali et al. (2021)	31	5 mL autologous microfat graft	AOFAS, FADI, VAS	Significant improvement at 6, 12, and 24 months	Autologous microfragmented adipose tissue is effective for the treatment of osteoarthritis pain in the ankle joints
Kim et al. (2016)	64	33 SMO + BMS, 31 SMO + MSC + BMS	VAS, AOFAS, TAS, TT, TLs, ICRS	VAS: preoperative 7.2 ± 1.1 (group 1) vs. 7.2 ± 0.8 (group 2) final follow-up 4.9 ± 1.3 vs. 3.7 ± 1.5.AOFAS score: preoperative 62.3 ± 6.1 vs. 61.0 ± 5.8 final follow-up 81.2 ± 6.2 vs. 85.2 ± 5.2TAS	Bone marrow stimulation is effective in patients with inversion ankle osteoarthritis undergoing SMO, and is better in combination with MSC injections
Kim and Koh (2016)	49	23BMS, 26 MSC + BMS	VAS, AOFAS, ICRS	Mean VAS scores improved from 7.3 ± 0.9 to 3.9 ± 1.2 in group 1 and from 7.4 ± 0.8 to 3.1 ± 1.5 in group 2 (P < 0.001 for both groups) Mean AOFAS scores also improved from 64.4 ± 4.1 to 79.6 ± 7.7 in group 1 and from 63.5 ± 4.2 to 84.2 ± 7.9 in group 2 (P < 0.001 for both groups) ICRS scores were higher in Group 2	Bone marrow stimulation is effective in patients with inversion ankle osteoarthritis undergoing lateral sliding osteotomy of the heel bone, and combined with MSC is more effective
Kim et al. (2014a)	50	26 BMS, 24 MSC + BMS	VAS, AOFAS, MRI MOCART, Tegner	Mean VAS score, AOFAS score ±1.2, 68.5 ± 5.6, and 3.4 ± improved from 7.1 1.2, 68.5, and 3.4 to 0.6 ± 0.8, 78.3 ± 4.9, and 3.5 ± 0.8, respectively, in the conventional group and from 7.1 ± 0.8, 67.7 ± 4.7, and 3.4 ± 0.5 to 3.2 ± 0.8, 83.3 ± 7.0, and 3.9 ± 0.7 in the MSC group. 7.0 and 3.9 ± 0.7 in the MSC group.	Bone marrow stimulation is effective in the treatment of cartilaginous lesions of the talus, and co-injection of MSC-containing SVF is more effective
Kim et al. (2013)	68	37BMS, 31 MSC + BMS	VAS, AODAS, Regner	VAS score improved significantly from 7.2 ± 1.1 to 4.0 ± 1.1 in group A and from 7.1 ± 1.0 to 3.2 ± 0.9 in group B. AOFAS score improved from 68.0 ± 5.5 to 77.2 ± 4.8 in group A and from 68.1 ± 5.6 to 82.6 ± 6.4 in group B. AOFAS scores improved from 68.0 ± 5.5 to 77.2 ± 4.8 in group A and from 68.1 ± 5.6 to 82.6 ± 6.4 in group B. Tegner Activity Scale scores improved significantly in group B	Bone marrow stimulation is effective for talar chondral lesions in patients over 50 years of age, and combined with MSCs is more effective, especially if the lesion is greater than 109 mm ooh the presence of subchondral cysts

#### 3.1.2 Ankle osteoarthritis

Ankle osteoarthritis (OA) is less common than knee and hip OA, with 75%–80% of clinical cases being trauma-related, typically resulting from ligament or bone injuries to the ankle. Conservative treatment for ankle OA currently focuses on pain relief, while surgical treatments for end-stage ankle OA are primarily centered around ankle arthrodesis and total ankle replacement (Anastasio et al., 2024).

##### 3.1.2.1 PRP

PRP injection therapy has been shown to improve the function and activity of ankle OA and has significant analgesic effects (Evans et al., 2020; Xiong et al., 2023), with particularly notable results in the short term (Laohajaroensombat et al., 2023). However, in long-term treatments (26 weeks (Paget et al., 2021) and 52 weeks (Paget et al., 2023)), the therapeutic effects do not show significant improvements.

##### 3.1.2.2 HA

HA injections improve the function of patients with ankle OA (Karatosun et al., 2008), but require long-term administration. A single intra-articular injection of low-molecular-weight, non-crosslinked hyaluronic acid does not show significant functional improvements (DeGroot et al., 2012). For long-term injections, a regimen of 25 mg sodium hyaluronate injected intra-articularly over 5 consecutive weeks can alleviate the signs and symptoms of ankle OA (Mei-Dan et al., 2010). Weekly intra-articular injections of sodium hyaluronate for 5 weeks also show good results (Salk et al., 2005). Injections of sodium hyaluronate with a molecular weight of 500–730 kDa are well tolerated (Salk et al., 2006), and studies suggest that clinical benefits can be observed as early as 1 week, potentially lasting for 6 months or longer (Sun et al., 2006). Additionally, injecting hyaluronic acid three times per week also provides excellent clinical outcomes (Sun et al., 2011).

The main information of Ankle Osteoarthritis included literature is shown in Table 6.

**TABLE 6:** Summary of papers on ankle osteoarthritis.

References	Sample size	Method	Scoring system	Results	Conclusion
Paget et al. (2021)	100	100 patients randomly assigned (1:1) to receive 2 intra-articular PRP injections (n = 48) or placebo (saline; n = 52).	AOFAS	The PRP group showed a 10-point increase in the American Orthopaedic Foot and Ankle Society (AOFAS) score (from 63 to 73 [95% CI, 6–14]; P < 0.001). The placebo group showed an 11-point increase	In ankle OA patients, intra-articular PRP injections did not significantly improve ankle pain symptoms over 26 weeks.
Paget et al. (2023)	100	100 ankle OA patients randomly assigned to PRP or placebo (saline) groups, receiving 2 intra-articular injections.	AOFAS	The unadjusted group difference in AOFAS scores at 52 weeks was 4 points (95% CI, −7 to −1; P = 0.02), favoring the placebo group.	PRP injections did not improve ankle pain symptoms over 52 weeks in ankle OA patients.
Karatosun et al. (2008)	43	43 ankle OA patients, with 30 randomly assigned to 3 intra-articular HA injections and 13 to weekly or exercise therapy for 6 weeks.	AOFAS	Both groups showed improvement in AOFAS scores. HA group: from 61.6 ± 16.8 to 90.1 ± 9.7, exercise group: from 72.1 ± 16.6 to 87.5 ± 17.5.	HA injections improve function in ankle OA.
DeGroot et al. (2012)	56	56 ankle OA patients, randomized to single intra-articular injection of 2.5 mL low-molecular-weight, non-crosslinked HA or saline solution.	AOFAS	AOFAS scores: HA group improved by 4.9 points at 6 and 12 weeks; placebo group worsened by 0.4 points at 6 weeks, then improved by 5.4 points at 12 weeks.	Single intra-articular injection of low-molecular-weight, non-crosslinked HA does not significantly improve function in ankle OA.
Mei-Dan et al. (2010)	16	16 arthritis ankle patients receiving 25 mg sodium hyaluronate injections for 5 consecutive weeks.	Visual Analog Scale and Ankle-Hindfoot Score	Range of motion improved by 20%, pain significantly reduced.	Sodium hyaluronate injections alleviate the signs and symptoms of ankle OA.
Salk et al. (2005)	17	Weekly intra-articular injections of sodium hyaluronate for 5 sessions.			Sodium hyaluronate intra-articular injections provide sustained pain relief and functional improvement in ankle OA.
Salk et al. (2006)	20	5weekly intra-articular injections of 1 mL sodium hyaluronate (10 mg/mL) or saline solution.	Ankle OA Score	In the sodium hyaluronate group, 5 out of 9 patients showed a >30 mm improvement in their scores.	Weekly intra-articular injections of sodium hyaluronate (500–730 kDa) provide good tolerance, sustained pain relief, and improved ankle OA function.
Sun et al. (2006)	75	75 patients receiving 5 weekly intra-articular HA injections.	AOS, AOFAS, ROM	AOS pain score: decreased from 4.8 ± 1.7 cm to 2.8 ± 2.0 cm (1 week), 2.1 ± 1.7 cm (1 month), and 2.1 ± 1.6 cm (3 months) (P < 0.001). AOFAS score increased from 64 ± 17 to 75 ± 15 (1 week), 78 ± 16 (1 month), and 78 ± 14 (3 months).	Intra-articular HA injections alleviate pain and improve function in ankle OA.
Sun et al. (2011)	46	46 patients receiving 3 weekly intra-articular sodium hyaluronate injections.	AOFAS	AOFAS scores improved from 60.5 at baseline to 73.5 (1 month), 75.5 (3 months), and 76.7 (6 months)	Weekly 3 injections of sodium hyaluronate improve pain in patients with unilateral ankle OA.

#### 3.1.3 Achilles tendon injury

Achilles tendon rupture (ATR) is the most common type of tendon rupture, accounting for 10.7% of all tendon and ligament injuries. In North America and Europe, the annual incidence of ATR ranges from 2.5 to 47 per 100,000 people (Briggs-Price et al., 2024). 60.1% of cases are secondary to sports-related mechanisms. Basketball is the most common sport-related mechanism, accounting for 36.6% of cases (Lyons et al., 2024). Heterotopic ossification (HO) occurs relatively frequently following Achilles tendon rupture. A clinical study indicated that nearly 20% of patients who underwent surgical repair of Achilles tendon rupture exhibited some degree of HO within the healing tendon (Sullivan et al., 2021). HO can lead to pain and functional impairment (Sullivan et al., 2021); however, it cannot be prevented through early rehabilitation (Guyton, 2020).

There is some evidence supporting the efficacy of a single PRP injection in treating chronic Achilles tendinopathy (AT). Monto (2012) treated 30 patients with chronic AT, who had failed 6 months of conservative treatment, with a single ultrasound-guided PRP injection. Three months post-treatment, the average AOFAS score improved from 34 to 92, with 88 points remaining at 24 months. Imaging showed resolution of Achilles tendon abnormalities in 27 of the 29 patients after 6 months. Clinical success was achieved in 28 of 30 patients. Krogh et al. (2016) studied 24 patients with a median disease duration of 33 months and treated them with blinded PRP (n = 12) or saline (n = 12). There were no differences in the VISA-A scores, but the PRP group showed tendon thickening. Boesen et al. (2017) indicated that PRP combined with eccentric training could reduce pain, improve activity levels, and reduce tendon thickness.

Regarding the long-term efficacy of PRP, repeated PRP injections show better outcomes. After three bi-weekly ultrasound-guided injections, the VISA-A score increased from baseline 49.9 ± 18.1 to 62.9 ± 19.8 at 2 months (Filardo et al., 2014).

However, the application of PRP in ATR patients shows limited effectiveness (Boesen et al., 2020; Schepull et al., 2011; Keene et al., 2022), and in non-insertional Achilles tendinopathy, the combination of endoscopic debridement with PRP shows limited results (Thermann et al., 2023).

#### 3.1.4 Plantar fasciitis

Plantar fasciitis is a common musculoskeletal injury, especially among runners, affecting approximately 17.4% of runners (Rhim et al., 2021). Risk factors include restricted ankle dorsiflexion, increased body mass index, and prolonged standing. Treatment should begin with stretching the plantar fascia, icing, and nonsteroidal anti-inflammatory drugs. Refractory plantar fasciitis may require injections, extracorporeal shock wave therapy, or surgical intervention (Trojian and Tucker, 2019).

##### 3.1.4.1 PRP

PRP injections are widely used in the treatment of plantar fasciitis, with studies assessing pain relief and reduction in fascia thickness using VAS, FADI, and AOFAS scores. Compared to corticosteroids, PRP treatment is more effective in relieving pain and restoring function, with longer-lasting effects (Shetty et al., 2019). For specific populations such as athletes, PRP injections can also accelerate functional recovery (Alessio-Mazzola et al., 2023).

##### 3.1.4.2 HA

HA is used as a non-surgical treatment for knee OA and persistent shoulder pain, and its anti-inflammatory effects hold promise for treating plantar fasciitis. Studies suggest that HA injections are a safe and effective conservative treatment option for plantar fasciitis, significantly alleviating pain and improving function, with no severe adverse events; mild injection site reactions resolve spontaneously (Kumai et al., 2014; Kumai et al., 2018). However, other studies have pointed out that HA is less effective than corticosteroids for short-term treatment of plantar fasciitis (Raeissadat et al., 2020). Regarding the choice between PRP and corticosteroids, Breton et al. (2022) and others suggest that the treatment method should be based on initial fascia thickness. For patients with an initial fascia thickness greater than 7 mm, corticosteroids are recommended. Tabrizi et al. (2020) suggest that for obese patients with a BMI ≥30 kg/m^2^, corticosteroids should be preferred.

##### 3.1.4.3 Hypertonic glucose

Hypertonic glucose injections mediate tissue repair through local initiation of wound healing phases (formation, inflammation, remodeling) and extracellular matrix synthesis (Kesikburun et al., 2022). Proposed mechanisms include VEGF pathway activation and cytokine modulation, though precise proliferative actions require further elucidation (Chutumstid et al., 2023).

Hypertonic glucose injection therapy is highly effective in treating plantar fasciitis (Kesikburun et al., 2022; Ersen, 2018; Kim et al., 2014b; Raissi et al., 2023; Ugurlar et al., 2018; Asheghan et al., 2021). Glucose prolotherapy has comparable efficacy to radial shock wave therapy in alleviating pain, improving daily function, and reducing plantar fascia thickness in plantar fasciitis patients (Kesikburun et al., 2022; Asheghan et al., 2021), although its effect is less pronounced than corticosteroid injection in the early stages (Raissi et al., 2023; Ugurlar et al., 2018).

##### 3.1.4.4 Placenta

In the treatment of plantar fasciitis, Sun XP et al. found that human placenta membrane, cryopreserved and intact, could serve as an adjunctive treatment and significantly relieve pain (Sun et al., 2018). Amnion is a component of the human placenta membrane. Werber (2015) found that a single injection of human amniotic tissue-amniotic fluid significantly alleviated pain caused by plantar fasciitis. Zelen et al. (2013) observed that micro-powdered dehydrated human amniotic membrane improved symptoms and function in chronic plantar fasciitis after 8 weeks. Hanselman et al. (2015) compared cryopreserved human amniotic membrane injections with corticosteroid treatment for plantar fasciitis. After 12 weeks of follow-up, both groups showed similar results on the Foot Health Status Questionnaire and VAS scores. Nakagawa et al. (2022) found that combining amniotic membrane allograft injections with ultrasound-guided percutaneous plantar fascia release resulted in better early pain relief.

The main information of Plantar Fasciitis included literature is shown in Table 7.

**TABLE 7:** Summary of papers on plantar fasciitis.

References	Sample size	Method	Scoring system	Results	Conclusion
Shetty et al. (2019)	90	90 patients randomized into PRP (n = 30), CS (n = 30), and placebo (n = 30) groups. Follow-up at 1 week, 3 weeks, and 3, 6, 12, and 18 months.	VAS, RM	The PRP group showed better long-term outcomes, with significant improvements in VAS (PRP: 8.2 to 2.1; CS: 8.8 to 3.6; placebo: 8.1 to 5.4) and RM scores (PRP: 1.7 to 3.7; CS: 1.2 to 3.1; placebo: 1.2–2.0).	PRP is more effective than CS for the long-term treatment of chronic plantar fasciitis, with better pain relief, functional outcomes, and lower need for further injections or surgery.
Alessio-Mazzola et al. (2023)	55	24 athletes among 55 subjects, all completed treatment. PRP group received 3 injections, ESW group received at least 3 treatments.	VAS, FFI	PRP group recovered faster (average 3.1 months, ESWT 5.9 months, p = 0.044) and had a lower overall recurrence rate (0% vs. 11.1%).	PRP may offer advantages over ESWT, especially for athletes, by reducing recurrence and promoting faster recovery of physical activity.
Kumai et al. (2014)	16	16 patients received up to 2.5 mL HA injection.	VAS	VAS score change for plantar fasciitis patients was −2.38 ± 2.61 cm.	HA provides safe pain relief for plantar fasciitis with no severe adverse events; minor injection site reactions resolved spontaneously.
Kumai et al. (2018)	168	Three groups of 56 patients each, receiving weekly injections of 2.5 mL 1% HA (H-HA), 0.8 mL 1% HA (L-HA), or 2.5 mL 0.01% HA (control) for 5 weeks.	VAS, Roles , Maudsley , ADLs	H-HA group showed significant improvement in VAS (−3.3 ± 0.3 cm) compared to control (−2.4 ± 0.3 cm, P = 0.029). Roles, Maudsley scores, and ADLs improved, especially in H-HA group, with no severe adverse events.	HA injection is a safe and effective conservative treatment for plantar fasciitis, significantly relieving pain and improving function.
Raeissadat et al. (2020)	75	38 patients received high-molecular weight HA (1 mL HA20 mg + 1 mL lidocaine 2%) or CS injection (1 mL methylprednisolone 40 mg + 1 mL lidocaine 2%) under ultrasound guidance.	VAS, FAAI , PPT , FFI , PFT	Both groups showed significant improvements in all parameters (P < 0.001). At 6 weeks, CS group showed more significant reduction in PFT and increased PPT (P = 0.004 and P = 0.011, respectively).	HA is not more effective than corticosteroids for short-term treatment of plantar fasciitis.
Breton et al. (2022)	38	PRP group (20) received 2 mL injections in tendons, CS group (18) received 1.5 mL injections around the tendon.	VAS, FFI, tendon thickness	CS group showed moderate correlation between fascia thickness and pain intensity (VAS), and total FFI score. PRP was effective regardless of fascia thickness.	PRP is effective regardless of fascia thickness, while corticosteroids work best in patients with greater fascia thickness.
Tabrizi et al. (2020)	31	16 obese patients received single CS injection, 15 received weekly PRP injections.	VAS, FFI	At 24 weeks, CS group showed greater pain relief and improvement in FFI	Corticosteroid injections are more effective than PRP for relieving pain and improving function in obese plantar fasciitis patients.
Kesikburun et al. (2022)	29	ESWT group (n = 15) or prolotherapy group (n = 14).	VAS, FFI, RMS	Both treatment groups showed significant improvement in VAS, RMS, and FFI scores at 6 and 12 weeks. No significant group differences at each time point.	ESWT and prolotherapy are equally effective for plantar fasciitis treatment.
Ersen (2018)	50	Prolotherapy group (n = 26) received 3 injections every 21 days, control group (n = 24) received stretching exercises for 3 months.	VAS , FAOS , FFI	Prolotherapy group showed better VAS, FAOS, and FFI scores at 42, 90, and 360 days compared to control group.	Prolotherapy is an effective adjunctive treatment for chronic plantar fasciitis.
Kim et al. (2014b)	20	PRP group (n = 9) received 2 injections of autologous PRP, DP group (n = 11) received 2 injections of 15% glucose/lidocaine solution.	FFI	PRP improved total FFI by 30.4%, compared to 15.1% for DP.	Both PRP and prolotherapy are effective for chronic recalcitrant plantar fasciitis.
Raissi et al. (2023)	40	20 patients received a single injection of 40 mg methylprednisolone, 20 patients received 20% glucose injection.	NRS, FAAM-A, FAAM-S	CS group showed significantly better NRS and FAAM-S scores compared to glucose prolotherapy at 2 weeks.	Both glucose prolotherapy and CS injections are effective for treating plantar fasciitis, but CS injections may be more effective in the early stages.
Ugurlar et al. (2018)	158	ESWT group (n = 39), prolotherapy group (n = 40), PRP group (n = 39), CS group (n = 40).	VAS, FFI-R	Prolotherapy and PRP groups showed significant VAS score reduction (p < 0.05) from 3 to 12 months. CS showed early effectiveness, but no significant differences at 36 months.	ESWT and prolotherapy have similar long-term outcomes, with PRP showing sustained effects; corticosteroids are most effective in the first 3 months.
Asheghan et al. (2021)	59	ESWT group (n = 29) and glucose injection group (n = 30) received treatments under ultrasound guidance.	VAS, FAAM	Both groups showed significant pain reduction at 6 and 12 weeks compared to baseline.	Glucose prolotherapy is as effective as ESWT in relieving pain and improving daily function in plantar fasciitis patients.
Sun et al. (2018)	1	A 53-year-old patient with chronic plantar fasciitis received cryopreserved human placenta membrane (vCPM) after conservative treatment failed.	NRS-11	Patient returned to full-time work with minimal symptoms at 12 and 24 months of follow-up.	Human placenta membrane can be an adjunctive treatment for recalcitrant plantar fasciitis.
Werber (2015)	44	ZimmerWave radial pulse treatment with ultrasound-guided injection of PalinGen SportFLOW and lidocaine.	VAS	Significant pain reduction reported at 4 weeks post-treatment, with average pain reduced to level 2 by week 12.	Cryopreserved amniotic membrane can be successfully used for treating chronic plantar fasciitis and Achilles tendonitis.
Zelen et al. (2013)	45	Control group (15) received standard care with 2 injections of Marcaine and saline, mDHACM group received 2 injections with mDHACM.	AOFAS, Wong–Baker FACES, SF-36v3	mDHACM group showed significant improvement in AOFAS, pain scores, and physical component scores compared to control.	mDHACM is a feasible treatment for refractory plantar fasciitis.
Hanselman et al. (2015)	23	14 patients randomly received corticosteroid injections, 9 received c-hAM injections.	VAS, FHSQ	At 6 weeks, corticosteroids showed better foot health scores, while c-hAM showed greater foot pain relief at 18 weeks.	Cryopreserved hAM injections are comparable to corticosteroid injections in treating plantar fasciitis.
Nakagawa et al. (2022)	31	UGPF group (n = 15) received surgery, UGPF + AM group (n = 16) received surgery plus AM injection.	VAS	UGPF + AM group showed better short-term pain relief, but no significant differences at 52 weeks.	AM injection combined with UGPF offers better short-term pain relief, with similar long-term outcomes.

#### 3.1.5 Ligament injury

Acute ankle injuries are among the most common musculoskeletal injuries and are often accompanied by intra-articular damage (Bsoul et al., 2024). Ankle sprains typically cause pain, leading to missed work and/or restricted daily activities, and may result in ankle instability and other functional impairments (Kemler et al., 2011). The ankle complex consists of the ankle joint (subtalar joint), the tibiofibular joint (talus), and the transverse tarsal joint (ankle joint). The Achilles tendon attaches to the calcaneus and provides stability to the tibiofibular joint through the interosseous tibiofibular ligament, lateral collateral ligament, anterior talofibular ligament, and the tibionavicular ligament of the deltoid ligament (Lp et al., 2023). For ligament injuries, conservative treatment can also be effective. Compared to conservative treatment, surgical intervention has not shown clear advantages in the repair of these ligaments, particularly in recovery time and complication rates, thus surgery is not recommended (Lim et al., 2019). PRP treatment has proven effective in alleviating pain and promoting functional recovery during the healing process of ankle injuries (Zhang et al., 2022). For grade II lateral ankle sprain patients, PRP treatment significantly reduced pain and promoted functional recovery at 8 weeks (Blanco-Rivera et al., 2020). In athletes with high-grade ankle sprains, PRP helps stabilize the syndesmosis joint and reduce long-term residual pain (Laver et al., 2015). For chronic ankle sprains, especially in chronic lateral ankle instability patients, PRP also shows significant efficacy, effectively improving symptoms and function (Medina-Porqueres et al., 2024), and two consecutive PRP injections may yield better outcomes (Zhang et al., 2022). However, the effectiveness of PRP for treating ankle injuries remains debated. Rowden et al. (2015) noted that PRP did not show significant efficacy in treating acute ankle sprains. Similarly, Sabaghzadeh et al. (2023) found that PRP did not significantly improve function when used post-MBG surgery.

### 3.2 Key technological platforms in regenerative medicine

This section outlines the key technological platforms underpinning regenerative medicine applications in the foot and ankle, ranging from established cell-based therapies to emerging engineering and computational approaches.

#### 3.2.1 Stem cell-based therapies

Stem cells form a cornerstone of regenerative medicine due to their defining capacities for self-renewal and multi-lineage differentiation. These cells are broadly categorized by their developmental potential: pluripotent stem cells (including embryonic and induced pluripotent stem cells), multipotent stem cells, and unipotent stem cells (Kimbrel and Lanza, 2020). While embryonic stem cells represent a source of natural pluripotency, their clinical application is constrained by ethical considerations and risks of immune rejection (Liu et al., 2020). Induced pluripotent stem cells, generated by reprogramming somatic cells, offer a patient-specific alternative but face challenges such as a bias toward fetal-state differentiation and incompletely defined molecular mechanisms.

In clinical practice for foot and ankle disorders, MSCs are the most widely utilized adult stem cell type. MSCs demonstrate the ability to differentiate into osteogenic, chondrogenic, and adipogenic lineages (Qin et al., 2023), and their therapeutic effect is further enhanced through the secretion of paracrine factors that modulate the local microenvironment (Pittenger et al., 1999). Common clinical sources of MSCs include:Bone Marrow: BMAC contains a heterogeneous mixture of cellular components, including platelets, monocytes, and MSCs, providing a rich regenerative milieu (Lan et al., 2021b). Adipose Tissue: Adipose-derived mesenchymal stem cells (ADSCs) possess multi-lineage differentiation potential and robust regenerative capabilities. Compared to bone marrow-derived MSCs, ADSCs offer advantages of higher accessibility, greater abundance, and lower donor site morbidity (Yuan et al., 2024). Placental and Amniotic Tissues: The human amniotic membrane (hAM) is considered an important source of stem cells (Igura et al., 2004; Hu et al., 2023) and contains multiple bioactive factors that synergistically promote tissue repair and regeneration (Parolini et al., 2009).

#### 3.2.2 3D printing

3D printing technology constructs objects layer by layer based on digital models and has been widely adopted in the biomedical field, particularly in personalized medicine. In the treatment of ankle disorders, its value is primarily demonstrated in: (1) fabricating customized implants for bone repair, such as talar prostheses (Giannotti et al., 2023; Gatz et al., 2020; Hannon et al., 2016); (2) producing physical anatomical models for preoperative planning of complex surgeries [72,147,148]; and (3) manufacturing patient-matched surgical guides and orthotic devices (Gormeli et al., 2015; Sun et al., 2018; Briggs et al., 2009) (e.g., AFOs). However, it must be noted that most existing studies supporting these applications are relatively small in scale, and many conclusions regarding the functional advantages of “personalized implants” are derived from case reports or small case series. Therefore, such claims should be interpreted with caution, and their clinical benefits require further validation. The general challenges facing the technology mainly involve printing accuracy and biomimeticity, material biocompatibility and regulatory compliance, as well as performance limitations of bioinks (Li et al., 2022). Specifically, in applications such as surgical guides, additional constraints include limited material options, suboptimal cost and time efficiency, insufficient long-term clinical evidence, and a lack of industry standards and regulations (Meng et al., 2022).

#### 3.2.3 Exosomes

Exosomes are nanoscale extracellular vesicles actively secreted by cells, with a diameter of 30–150 nm, encased in a lipid bilayer membrane, containing proteins, nucleic acids, and other active molecules. The production process involves cell endocytosis, forming early endosomes, which mature into multivesicular bodies under the action of endosomal sorting complexes, eventually being released extracellularly. Exosomes play a critical role in intercellular communication, substance transport, immune regulation, tumor development, and tissue repair and regeneration (Doyle and Wang, 2019; Zhang et al., 2015). Although exosomes theoretically offer numerous benefits, research on exosomes remains confined to laboratory animal models. Exosome-based therapies for ankle repair show immense promise, leveraging their ability to promote osteogenesis, angiogenesis, and chondrogenesis. For instance, exosomes derived from mesenchymal stem cells (MSCs) and other sources enhance tissue repair by delivering key regulatory molecules (e.g., miR-126, miR-140-5p) (Yu et al., 2021; Jiang et al., 2020; Behera et al., 2021). (Wu et al., 2019; Liu et al., 2018; Zhang et al., 2018). However, the transition from promising research to clinical application faces significant hurdles. Key challenges include the lack of standardized methods for exosome isolation, quantification, and drug loading, which impacts batch-to-batch consistency and therapeutic reproducibility (Rezaie et al., 2022; Tian et al., 2023). Furthermore, critical translational barriers must be addressed, such as establishing clear regulatory pathways (e.g., FDA/EMA classification as a biologic or drug product), implementing Good Manufacturing Practice (GMP) compliance for production, ensuring quality control, and navigating ethical requirements for informed consent and long-term patient follow-up in clinical trials.

#### 3.2.4 Artificial intelligence

AI has emerged as a pivotal enabler in the advancement of regenerative medicine. Central to this role are machine learning—which identifies patterns from datasets—and deep learning—which excels at processing complex, high-dimensional data. Together, these methodologies constitute the analytical foundation of predictive regenerative medicine. AI is transforming the field across several critical fronts: it significantly enhances the discovery, development, and manufacturing of biotherapeutics, including cell and gene therapies, while also providing robust decision support in clinical trial design, patient stratification, and dynamic treatment assessment. These advances collectively contribute to improved diagnostic accuracy, research and development (R&D) efficiency, and therapeutic outcomes. However, the predictive performance and generalizability of AI models are heavily contingent upon the quality and comprehensiveness of the training data. Consequently, the successful implementation of such approaches necessitates unprecedented levels of accuracy, standardization, and consistency in multi-source clinical data—encompassing genomic profiles, medical histories, and imaging data—obtained from collaborative research and clinical centers. Inadequate data governance and quality control remain significant impediments to the widespread adoption of this research paradigm (Garmany and Terzic, 2024).

## 4 Discussion

This scoping review synthesizes regenerative medicine advancements for five major foot/ankle pathologies: ligament injuries, talar cartilage defects, ankle osteoarthritis, Achilles tendinopathy, and plantar fasciitis. Although significant methodological variations and ongoing debates regarding efficacy exist, current evidence indicates a trend toward the clinical potential of regenerative therapies in areas such as pain relief, functional recovery, and tissue repair. Nonetheless, the observed heterogeneity among studies highlights the need for further rigorously designed trials.

PRP is an autologous blood-derived product processed to concentrate platelets and related growth factors (Carr, 2022). PRP therapy is based on platelet-derived growth factors that support the three stages of wound healing and repair: inflammation, proliferation, and remodeling (Everts et al., 2020). PRP contains a large number of growth factors and cytokines that promote tissue regeneration, accelerate wound healing, and reduce inflammation. Due to its theoretical potential to repair tissues with poor healing capabilities, PRP is increasingly used in the treatment of various musculoskeletal diseases (Martinez-Martinez et al., 2018). In addition to PRP, platelet derivatives such as platelet-rich fibrin (PRF) and concentrated growth factors (CGF) have different clinical effects (Giannotti et al., 2023). However, the translation of this strong theoretical foundation into consistent clinical evidence faces significant challenges. Current evidence on PRP therapy in the foot and ankle field indicates that the methodological quality of randomized controlled trials is generally low to moderate (Bennell et al., 2017), with limitations such as inadequate blinding and small sample sizes. Therefore, conclusions from any single study should be interpreted with caution. The core issue has shifted from “whether PRP is effective” to “how to define and standardize PRP to enable meaningful comparisons and optimization” (Kieb et al., 2017). Considerable variations exist across studies regarding the number and timing of injections. Some researchers adopt repeated injection protocols based on the rationale of sustaining growth factor release to enhance efficacy (Filardo et al., 2014). Such fundamental discrepancies in treatment protocols lead to inconsistent conclusions and pose challenges for evidence synthesis. Moreover, the efficacy of PRP is influenced by multiple factors. First, platelet concentration has an “optimal window”: insufficient concentration may fail to deliver adequate growth factors (e.g., TGF-β1, PDGF), while excessive concentration may trigger exaggerated inflammatory responses and impair healing (Sánchez-González et al., 2012). Second, leukocytes exhibit a complex dual role in PRP. On one hand, they help remove necrotic tissue and secrete specific cytokines to initiate repair; on the other hand, excessive activation may release pro-inflammatory mediators, aggravating local inflammation and potentially leading to adverse outcomes (Dohan Ehrenfest et al., 2012). Furthermore, leukocyte content affects the release kinetics of growth factors: high-leukocyte PRP tends to form more stable fibrin scaffolds enabling sustained release, which may be more suitable for chronic injuries; whereas low-leukocyte PRP is characterized by a rapid, burst-like release of growth factors (Calciolari et al., 2025). Finally, the preparation technique (e.g., centrifugation conditions) profoundly influences the structure of the fibrin matrix and the release kinetics of growth factors, thereby modulating the overall biological effects of PRP (Giannotti et al., 2023). Even the use of activators (e.g., Ca^2+^) is not merely for accelerating activation but also regulates the release pattern of growth factors (Steller et al., 2019). Therefore, there is currently no universally accepted standardized protocol for PRP. Its definition remains ambiguous, encompassing a range of unresolved variables such as platelet and leukocyte concentrations, activation methods and agents, anticoagulant use, and final product form (Anitua et al., 2022). In the treatment of foot and ankle disorders (such as Achilles tendinopathy, ligament injuries, and osteoarthritis),PRP represents an emerging biological therapy. However, it must be clearly recognized that PRP is not a single, standardized “drug.” Therefore, in the absence of specific clinical practice guidelines for PRP, management should adhere to existing general guidelines. We anticipate that more standardized, high-quality studies will emerge in the future to refine its treatment protocols and provide more definitive and reliable conclusions for clinical practice.

HO is an aberrant regenerative process characterized by the pathological deposition of bone tissue within soft tissues where it does not normally occur, such as tendon regions following Achilles tendon rupture (Lin et al., 2010; Lui et al., 2009). The precise mechanisms underlying its formation remain incompletely elucidated; it is generally regarded as a pathological wound healing response subsequent to musculoskeletal trauma, involving both local and systemic inflammatory processes (Kraft et al., 2016). This process is potentially associated with the differentiation of stem or tendon cells into chondrocytes, followed by chondral hypertrophy and calcification, ultimately leading to osteogenesis (Lui et al., 2009). Li and Tuan (2020) proposed that muscle injury-induced upregulation of local BMP-7 levels, combined with a systemic downregulation of TGF-β1 caused by glucocorticoid excess, may represent a critical pathogenic mechanism in traumatic HO (tHO) formation. Current management of traumatic HO remains predominantly prophylactic. Conventional strategies include nonsteroidal anti-inflammatory drugs (NSAIDs, e.g., celecoxib, which has been demonstrated to inhibit HO formation in rat models (Zhang et al., 2013) and glucocorticoids. However, celecoxib is ineffective against the progression of already initiated HO (Li et al., 2019). Recent investigations have revealed that a synergistic strategy combining engineered exosomes with 3D-printed scaffolds shows significant promise. This approach primarily entails (Xu et al., 2025): (1) pre-treating the cell source with a BMP signaling pathway inhibitor (e.g., LDN-193189) to endow the secreted exosomes with intrinsic “anti-osteogenic” activity; (2) engineering the exosomal membrane (e.g., by incorporating RGD peptides) to enhance its targeting capability and retention at the injury site; and (3) loading the modified exosomes onto a biodegradable 3D-printed sustained-release scaffold, which provides structural support while enabling the long-term controlled release of bioactive molecules. In rigorous animal models, this strategy not only significantly promoted tendon-like tissue regeneration and the restoration of biomechanical function but also, more importantly, effectively inhibited heterotopic ossification formation as confirmed by micro-CT and histological analyses.

The clinical translation of stem cells faces several challenges, including the need to optimize cell sources, differentiation protocols, and delivery methods to ensure treatment safety, efficacy, and reproducibility. Furthermore, post-transplant immune rejection responses and other potential adverse events require rigorous evaluation and long-term monitoring (Rahimi Darehbagh et al., 2024).
