# Supplementary material for: Applications of bone regenerative medicine in the foot and ankle: mechanisms, technologies, and therapeutic advances
Source: Front Bioeng Biotechnol. 2025 Dec 2;13:1653964. doi: 10.3389/fbioe.2025.1653964 (PMC12704982; doi:10.3389/fbioe.2025.1653964)
Supplement: Supplementary file 1 [file DataSheet2.pdf]

Molecular weight
Not provided
Cross - linked high molecular weight (Hylan G - F 20, Synvisc®)
25 mg/2.5 mL (ARTZ)
Half - dose (12.5 mg/1.25 mL)
2.5 mg/injection
620 - 1170 kDa (Supartz®)
600 - 1200 kDa (Adant®)
500 - 730 kDa (Hyalgan®)
500 - 730 kDa (Hyalgan®)
6.2 - 12 million Da
500 - 730 kDa (Hyalgan®)
2700 kDa (Suvenyl®)
2700 kDa (H - HA) vs 8 kDa (L - HA) (Suvenyl®)
>2000 kDa (Viscor®)

| Cross-linking status | Patient phenotype |
|---|---|
| Not provided | n = 15, age 42.8 ± 18.1 years, talus OCL |
| Cross - linked | n = 14, age 39.7 ± 8.7 years, talus OCL |
| Not mentioned | n = 18, age 34.7 ± 8.7 years, talus OCL |
| Not mentioned | n = 57, age 40.5 ± 13.0 years, talus OCL |
| Not mentioned | n = 15, ankle OA |
| Non - cross - linked | n = 39, age 54.1 - 61.9 years, ankle OA |
| Not mentioned | n = 16, age 43 years, ankle OA |
| Not mentioned | n = 9, age 57.8 - 60 years, ankle OA |
| Not mentioned | n = 17, age 57.8 - 60 years, ankle OA |
| Not mentioned | n = 75, age 50.2 ± 14.3 years, ankle OA |
| Not mentioned | n = 46, age 51.7 ± 14.4 years, ankle OA |
| Not mentioned | n = 61, age 43.5 years, enthesopathy |
| Not mentioned | n = 107, mean age 50.4 years, plantar fasciitis |
| Not mentioned | n = 38, age 41.73 ± 7.68 years, plantar fasciitis |

| Treating diseases | Safety |
|---|---|
| Talus Cartilage Injuries | No infection; mild pain |
| Talus Cartilage Injuries | No serious adverse events |
| Talus Cartilage Injuries | No complications |
| Talus Cartilage Injuries | No postoperative complications |
| Ankle Osteoarthritis | No HA - related complications |
| Ankle Osteoarthritis | One mild adverse event |
| Ankle Osteoarthritis | No infection; one case of mild pain |
| Ankle Osteoarthritis | No serious adverse events |
| Ankle Osteoarthritis | No serious adverse events |
| Ankle Osteoarthritis | Five cases of transient injection - site pain |
| Ankle Osteoarthritis | No serious adverse events |
| Plantar Fasciitis | No serious adverse events |
| Plantar Fasciitis | Mild injection - site pain |
| Plantar Fasciitis | No serious adverse events |

References

22253252
25825393
25763853
22205098
18565251
22218376
20237359
16213381
16452740
16635582
21938376
24817495
28255655
32021400
