# Supplementary material for: Applications of bone regenerative medicine in the foot and ankle: mechanisms, technologies, and therapeutic advances
Source: Front Bioeng Biotechnol. 2025 Dec 2;13:1653964. doi: 10.3389/fbioe.2025.1653964 (PMC12704982; doi:10.3389/fbioe.2025.1653964)
Supplement: Supplementary file 2 [file DataSheet4.pdf]

Cross-linking status	Patient phenotype	Treating diseases	Safety	References
Not provided	n = 15, age 42.8 ± 18.1 years, talus OCL	Talus Cartilage Injuries	No infection; mild pain	22253252
Cross - linked	n = 14, age 39.7 ± 8.7 years, talus OCL	Talus Cartilage Injuries	No serious adverse events	25825393
Not mentioned	n = 18, age 34.7 ± 8.7 years, talus OCL	Talus Cartilage Injuries	No complications	25763853
Not mentioned	n = 57, age 40.5 ± 13.0 years, talus OCL	Talus Cartilage Injuries	No postoperative complications	22205098
Not mentioned	n = 15, ankle OA	Ankle Osteoarthritis	No HA - related complications	18565251
Non - cross - linked	n = 39, age 54.1 - 61.9 years, ankle OA	Ankle Osteoarthritis	One mild adverse event	22218376
Not mentioned	n = 16, age 43 years, ankle OA	Ankle Osteoarthritis	No infection; one case of mild pain	20237359
Not mentioned	n = 9, age 57.8 - 60 years, ankle OA	Ankle Osteoarthritis	No serious adverse events	16213381
Not mentioned	n = 17, age 57.8 - 60 years, ankle OA	Ankle Osteoarthritis	No serious adverse events	16452740
Not mentioned	n = 75, age 50.2 ± 14.3 years, ankle OA	Ankle Osteoarthritis	Five cases of transient injection - site pain	16635582
Not mentioned	n = 46, age 51.7 ± 14.4 years, ankle OA	Ankle Osteoarthritis	No serious adverse events	21938376
Not mentioned	n = 61, age 43.5 years, enthesopathy	Plantar Fasciitis	No serious adverse events	24817495
Not mentioned	n = 107, mean age 50.4 years, plantar fasciitis	Plantar Fasciitis	Mild injection - site pain	28255655
Not mentioned	n = 38, age 41.73 ± 7.68 years, plantar fasciitis	Plantar Fasciitis	No serious adverse events	32021400
