# Supplementary material for: Applications of bone regenerative medicine in the foot and ankle: mechanisms, technologies, and therapeutic advances
Source: Front Bioeng Biotechnol. 2025 Dec 2;13:1653964. doi: 10.3389/fbioe.2025.1653964 (PMC12704982; doi:10.3389/fbioe.2025.1653964)
Supplement: Supplementary file 3 [file Table1.pdf]

- *Supplemental Table 1 List of abbreviations*

Unit	abbreviation
Mesenchymal Stem Cells	MSCs
Platelet-Rich Plasma	PRP
Bone Marrow Aspirate Concentrate	BMAC
Adipose-Derived Mesenchymal Stem Cells	ADSCs
Human Amniotic Membrane	hAM
Platelet-Rich Fibrin	PRF
Concentrated Growth Factors	CGF
Hyaluronic Acid	HA
Human Adipose-Derived Stem Cells	hASC
American Orthopedic Foot & Ankle Society	AOFAS
Foot Health Status Questionnaire	FHSQ
Visual Analog Scale	VAS
Magnetic Resonance Observation of Cartilage Repair Tissue	MOCART
Victorian Institute of Sports Assessment - Achilles	VISA-A
EuroQol Visual Analog Scale	EQ-VAS
Ankle Osteoarthritis	OA
Heterotopic ossification	HO
