# Supplementary material for: Applications of bone regenerative medicine in the foot and ankle: mechanisms, technologies, and therapeutic advances
Source: Front Bioeng Biotechnol. 2025 Dec 2;13:1653964. doi: 10.3389/fbioe.2025.1653964 (PMC12704982; doi:10.3389/fbioe.2025.1653964)
Supplement: Supplementary file 4 [file Table2.pdf]

- *Supplemental Table 2 Comparison of Platelet Derivatives*

Characteristics	PRP	PRF	CGF
Preparation Method	Double centrifugation, using anticoagulants	Single low-speed centrifugation, no anticoagulants	Alternating speed centrifugation, no anticoagulants
Platelet Concentration	3-5 times normal plasma	4-8 times normal plasma	15.5-17.8 times normal plasma
Growth Factor Release	Rapid release, within 1 hour	Sustained release, 7-10 days	Long-term sustained release, up to 28 days
Components	High concentration of platelets, some white blood cells	High concentration of platelets, white blood cells, fibrin	High concentration of platelets, white blood cells, stem cell-like cells
Main Applications	Sports medicine, plastic surgery, dentistry	Dentistry, orthopedics, oral surgery	Bone defect repair, ear/nose reconstruction, implants, nerve regeneration
Advantages	Simple, rapid preparation, widely used	Sustained growth factor release, no exogenous coagulation agents	Efficient and sustained growth factor release, supports multi-phase regeneration
Disadvantages	Short growth factor release time	Limited growth factor release amount and duration	Complex preparation process, requires specific equipment
