# Supplementary material for: Applications of bone regenerative medicine in the foot and ankle: mechanisms, technologies, and therapeutic advances
Source: Front Bioeng Biotechnol. 2025 Dec 2;13:1653964. doi: 10.3389/fbioe.2025.1653964 (PMC12704982; doi:10.3389/fbioe.2025.1653964)
Supplement: Supplementary file 5 [file DataSheet3.pdf]

PMID	22253252	25825393	24292979	34698782	37417359	22735279
Treating diseases	Talus Cartilage Injuries	Talus Cartilage Injuries	Talus Cartilage Injuries	Ankle Osteoarthritis	Ankle Osteoarthritis	Achilles Tendon Injury
Safety	No infection; a few cases of mild pain	No serious adverse events	1 case of transient nerve palsy, no infection	No infection or hematoma	No adverse events within 52 weeks	No complications
Patient phenotype	n = 15, aged 42.8 ± 18.1 years, ankle pain with OCL of the talus	n = 13, aged 38.6 ± 9.1 years, ankle pain with OCL of the talus	n = 19, aged 38.5 ± 12.7 years, ankle pain with OCL of the talus	n = 48, aged 55.6 ± 13.8 years, ankle osteoarthritis (OA)	n = 48, aged 54.8 ± 13.3 years, ankle OA	n = 30, aged 47 years, chronic Achilles tendinopathy
Number of injections	3 injections, 2 weeks apart	Injection 24 - 48 h after surgery, arthroscopic debridement + microfracture	Injection 6 - 24 h after surgery, after microfracture	2 injections, 6 weeks apart	2 injections, 6 weeks apart	Single injection, chronic Achilles tendinopathy
Preparation method	Centrifugation at 640g for 8 min, activated with $CaCl_2$	Smart PReP®2 system	Smart PReP®2 system	Arthrex dual-syringe system	Arthrex dual-syringe system	Accelerate system
Platelet concentration	2 - 3 times the baseline	236,000 → 1,227,000/μL	5.4 - fold increase, 1,335,500 /μL	Not analyzed	Not provided	7 - 10 times the baseline
Leukocyte content	Not mentioned	Not mentioned	Not mentioned	Leukocyte-poor	Not mentioned	Not mentioned
Volume	2 mL	Not specified	Not specified	2 mL	2 mL	2 mL on each side of the Achilles tendon
Activation method	Activated with $CaCl_2$	Not mentioned	Not mentioned; pH adjusted with $NaHCO_3$	Not activated	Not mentioned	Not activated

PMID	27257167	28530451	24960641	32485112	36317349	21051425
Treating diseases	Achilles Tendon Injury	Achilles Tendon Injury	Achilles Tendon Injury	Achilles Tendon Injury	Achilles Tendon Injury	Achilles Tendon Injury
Safety	No infection; 1 case of increased pain	No infection, hematoma or rupture	No serious adverse events	No injection-related side effects	No re-rupture	2 cases excluded (re-rupture, infection)
Patient phenotype	n = 12, aged 46.7 ± 9.0 years, Achilles tendinopathy	n = 19, aged 43.1 ± 8.1 years, chronic Achilles tendinopathy	n = 27, aged 44.6 ± 10.6 years, chronic Achilles tendinopathy	n = 40, male, acute Achilles tendon rupture	n = 182, aged 46 ± 13.0 years, acute Achilles tendon rupture	n = 16, aged 39.8 ± 6.2 years, acute Achilles tendon rupture
Number of injections	Single injection	4 injections, 2 weeks apart, chronic Achilles tendinopathy	3 injections, 2 weeks apart, chronic Achilles tendinopathy	4 injections, 2 weeks apart	Single injection, acute Achilles tendon rupture	Intra-operative injection of 10 mL, acute Achilles tendon rupture
Preparation method	Recover GPS II system	Arthrex dual-syringe system	Double centrifugation	Arthrex dual-syringe system	MAG 200 MAGELLAN system + MDK 300/300-1 kit	Double centrifugation
Platelet concentration	8 times the baseline	2.5 times the baseline	5 times the baseline	2.5 times the baseline	4.1 times the baseline	10 times the baseline
Leukocyte content	Not mentioned	Not mentioned	Leukocyte count 1.2 times the normal blood value	Not mentioned	Leukocyte count 2.2 times the normal blood value	Not mentioned
Volume	6 mL	4 mL	5 mL	4 mL	4 mL	10 mL
Activation method	Not mentioned; buffered with $NaHCO_3$	Not activated	Activated with $CaCl_2$	Not activated	Not activated	Activated with $CaCl_2$
PMID	32619656	30448183	36396549	21395362	36619392	31640921

Treating diseases	Achilles Tendon Injury	Plantar Fasciitis	Plantar Fasciitis	Ligament Injury	Ligament Injury	Ligament Injury
Safety	5 cases of mild complications	No complications	No adverse reactions	Not provided	Not mentioned	No complications
Patient phenotype	n = 17, aged 52.8 ± 9.8 years, Achilles tendinopathy	n = 90, aged 44.6 years, chronic plantar fasciitis	n = 28, aged 59.4 ± 12.0 years, chronic plantar fasciitis	Not provided	n = 27, aged 31.9 ± 8.2 years, ankle sprain	n = 11, aged 27.9 ± 12.1 years, ankle sprain
Number of injections	Intra-operative injection, after endoscopic debridement	Ultrasound-guided injection	3 injections per week	Not provided	1 or 2 injections	Single injection
Preparation method	Arthrex dual-syringe system	Standard double centrifugation	Triple centrifugation	Not provided	WEGO PRP kit	Double centrifugation
Platelet concentration	Not specified	Not specified	2 - 3 times the baseline	Not provided	> 6 times the baseline	Not specified
Leukocyte content	Not mentioned	Not mentioned	Low leukocyte count (<1000/mL)	Not provided	Leukocyte count ≤ 4 times the normal blood value	Not mentioned
Volume	Not specified	2 mL	3 mL	Not provided	3 - 4 mL	5 mL
Activation method	Not mentioned	Not mentioned	Not activated	Not provided	Activated with $CaCl_2$	Activated with calcium gluconate

PMID	24938396	26048069	37124365	34844893	31882151
Treating diseases	Ligament Injury	Ligament Injury	Ligament Injury	Plantar Fasciitis	Plantar Fasciitis
Safety	No re-injury	No adverse events	No side effects	No adverse events	No serious adverse events, 1 case excluded

					due to loss of follow-up
Patient phenotype	n = 8, aged 22.6 ± 4.2 years, ankle sprain	n = 18, aged 30.3 years, ankle sprain	n = 20, aged 34.45 ± 11.72 years, ankle instability	n = 20, aged 51 ± 11 years, chronic plantar fasciitis	n = 15, aged 33.6 ± 8.5 years, chronic plantar fasciitis
Number of injections	2 injections, 7 days apart	Single injection	3 injections	Single injection	3 injections per week
Preparation method	Centrifugation at 460g for 8 min, activated with $CaCl_2$	Magellan kit	Rooyagen kit	Double centrifugation (GPS III Biomet kit)	Arthrex dual-syringe system
Platelet concentration	29 - 39 times the baseline	Not provided	5 ± 1 times the baseline	Not provided	Not provided
Leukocyte content	No leukocytes	Not mentioned	Leukocyte-rich	Not mentioned	Not mentioned
Volume	1.5 mL	3 - 4 mL	2 mL for the first time; 4 mL each for the 2nd and 3rd times	2 mL	3 mL
Activation method	Activated with $CaCl_2$	Not mentioned	Not mentioned		
