# Supplementary material for: Applications of bone regenerative medicine in the foot and ankle: mechanisms, technologies, and therapeutic advances
Source: Front Bioeng Biotechnol. 2025 Dec 2;13:1653964. doi: 10.3389/fbioe.2025.1653964 (PMC12704982; doi:10.3389/fbioe.2025.1653964)
Supplement: Supplementary file 6 [file DataSheet1.pdf]

| Preparation method |
| --- |
| Centrifugation at 640g for 8 min, activated with $CaCl_2$ |
| Smart PReP®2 system |
| Smart PReP®2 system |
| Arthrex dual - syringe system |
| Arthrex dual - syringe system |
| Accelerate system |
| Recover GPS II system |
| Arthrex dual - syringe system |
| Double centrifugation |
| Arthrex dual - syringe system |
| MAG 200 MAGELLAN system + MDK 300/300 - 1 kit |
| Double centrifugation |
| Arthrex dual - syringe system |
| Standard double centrifugation |
| Triple centrifugation |
| Not provided |
| WEGO PRP kit |
| Double centrifugation |
| Centrifugation at 460g for 8 min, activated with $CaCl_2$ |
| Magellan kit |
| Rooyagen kit |
| Double centrifugation (GPS III Biomet kit) |
| Arthrex dual - syringe system |

| Platelet concentration |
| --- |
| 2 - 3 times the baseline |
| 236,000 → 1,227,000/μL |
| 5.4 - fold increase, 1,335,500/μL |
| Not analyzed |
| Not provided |
| 7 - 10 times the baseline |
| 8 times the baseline |
| 2.5 times the baseline |
| 5 times the baseline |
| 2.5 times the baseline |
| 4.1 times the baseline |
| 10 times the baseline |
| Not specified |
| Not specified |
| 2 - 3 times the baseline |
| Not provided |
| ＞ 6 times the baseline |
| Not specified |
| 29 - 39 times the baseline |
| Not provided |
| 5 ± 1 times the baseline |
| Not provided |
| Not provided |

Leukocyte content
Not mentioned
Not mentioned
Not mentioned
Leukocyte - poor
Not mentioned
Not mentioned
Not mentioned
Not mentioned
Leukocyte count 1.2 times the normal blood value
Not mentioned
Leukocyte count 2.2 times the normal blood value
Not mentioned
Not mentioned
Not mentioned
Low leukocyte count (<1000/mL)
Not provided
Leukocyte count ≤ 4 times the normal blood value
Not mentioned
No leukocytes
Not mentioned
Leukocyte - rich
Not mentioned
Not mentioned

| Volume |
| --- |
| 2 mL |
| Not specified |
| Not specified |
| 2 mL |
| 2 mL |
| 2 mL on each side of the Achilles tendon |
| 6 mL |
| 4 mL |
| 5 mL |
| 4 mL |
| 4 mL |
| 10 mL |
| Not specified |
| 2 mL |
| 3 mL |
| Not provided |
| 3 - 4 mL |
| 5 mL |
| 1.5 mL |
| 3 - 4 mL |
| 2 mL for the first time; 4 mL each for the 2nd and 3rd times |
| 2 mL |
| 3 mL |

| Activation method |
| --- |
| Activated with $CaCl_2$ |
| Not mentioned |
| Not mentioned; pH adjusted with $NaHCO_3$ |
| Not activated |
| Not mentioned |
| Not activated |
| Not mentioned; buffered with $NaHCO_3$ |
| Not activated |
| Activated with $CaCl_2$ |
| Not activated |
| Not activated |
| Activated with $CaCl_2$ |
| Not mentioned |
| Not mentioned |
| Not activated |
| Not provided |
| Activated with $CaCl_2$ |
| Activated with calcium gluconate |
| Activated with $CaCl_2$ |
| Not mentioned |
| Not mentioned |
| Not mentioned |
| Not mentioned |

injections: single vs repeated injections, surgical adjuncts (e.g., microfracture
3 injections, 2 weeks apart
Injection 24 - 48 h after surgery, arthroscopic debridement + microfracture
Injection 6 - 24 h after surgery, after microfracture
2 injections, 6 weeks apart
2 injections, 6 weeks apart
Single injection, chronic Achilles tendinopathy
Single injection
4 injections, 2 weeks apart, chronic Achilles tendinopathy
3 injections, 2 weeks apart, chronic Achilles tendinopathy
4 injections, 2 weeks apart
Single injection, acute Achilles tendon rupture
Intra - operative injection of 10 mL, acute Achilles tendon rupture
Intra - operative injection, after endoscopic debridement
Ultrasound - guided injection
3 injections per week
Not provided
1 or 2 injections
Single injection
2 injections, 7 days apart
Single injection
3 injections
Single injection
3 injections per week

| Patient phenotype |
| --- |
| 15, aged 42.8 ± 18.1 years, ankle pain with osteochondral lesion (OCL) of the ta |
| n = 13, aged 38.6 ± 9.1 years, ankle pain with OCL of the talus |
| n = 19, aged 38.5 ± 12.7 years, ankle pain with OCL of the talus |
| n = 48, aged 55.6 ± 13.8 years, ankle osteoarthritis (OA) |
| n = 48, aged 54.8 ± 13.3 years, ankle OA |
| n = 30, aged 47 years, chronic Achilles tendinopathy |
| n = 12, aged 46.7 ± 9.0 years, Achilles tendinopathy |
| n = 19, aged 43.1 ± 8.1 years, chronic Achilles tendinopathy |
| n = 27, aged 44.6 ± 10.6 years, chronic Achilles tendinopathy |
| n = 40, male, acute Achilles tendon rupture |
| n = 182, aged 46 ± 13.0 years, acute Achilles tendon rupture |
| n = 16, aged 39.8 ± 6.2 years, acute Achilles tendon rupture |
| n = 17, aged 52.8 ± 9.8 years, Achilles tendinopathy |
| n = 90, aged 44.6 years, chronic plantar fasciitis |
| n = 28, aged 59.4 ± 12.0 years, chronic plantar fasciitis |
| Not provided |
| n = 27, aged 31.9 ± 8.2 years, ankle sprain |
| n = 11, aged 27.9 ± 12.1 years, ankle sprain |
| n = 8, aged 22.6 ± 4.2 years, ankle sprain |
| n = 18, aged 30.3 years, ankle sprain |
| n = 20, aged 34.45 ± 11.72 years, ankle instability |
| n = 20, aged 51 ± 11 years, chronic plantar fasciitis |
| n = 15, aged 33.6 ± 8.5 years, chronic plantar fasciitis |

| Treating diseases |
| --- |
| Talus Cartilage Injuries |
| Talus Cartilage Injuries |
| Talus Cartilage Injuries |
| Ankle Osteoarthritis |
| Ankle Osteoarthritis |
| Achilles Tendon Injury |
| Achilles Tendon Injury |
| Achilles Tendon Injury |
| Achilles Tendon Injury |
| Achilles Tendon Injury |
| Achilles Tendon Injury |
| Achilles Tendon Injury |
| Achilles Tendon Injury |
| Plantar Fasciitis |
| Plantar Fasciitis |
| Ligament Injury |
| Ligament Injury |
| Ligament Injury |
| Ligament Injury |
| Ligament Injury |
| Ligament Injury |
| Plantar Fasciitis |
| Plantar Fasciitis |

Safety
No infection; a few cases of mild pain
No serious adverse events
1 case of transient nerve palsy, no infection
No infection or hematoma
No adverse events within 52 weeks
No complications
No infection; 1 case of increased pain
No infection, hematoma or rupture
No serious adverse events
No injection - related side effects
No re - rupture
2 cases excluded (re - rupture, infection)
5 cases of mild complications
No complications
No adverse reactions
Not provided
Not mentioned
No complications
No re - injury
No adverse events
No side effects
No adverse events
No serious adverse events, 1 case excluded due to loss of follow - up

References


22253252
25825393
24292979
34698782
37417359
22735279
27257167
28530451
24960641
32485112
36317349
21051425
32619656
30448183
36396549
21395362
36619392
31640921
24938396
26048069
37124365
34844893
31882151
