# Supplementary material for: Applications of bone regenerative medicine in the foot and ankle: mechanisms, technologies, and therapeutic advances
Source: Front Bioeng Biotechnol. 2025 Dec 2;13:1653964. doi: 10.3389/fbioe.2025.1653964 (PMC12704982; doi:10.3389/fbioe.2025.1653964)
Supplement: Supplementary file 7 [file DataSheet5.pdf]

PMID	25662594	27994720	30321966	35203279	34786970	30424945
Source	BMAC (iliac aspiration)	mBMAC vs mACI	BMAC	BMAC	BMAC	BMAC
Cell counts	60 mL of bone marrow concentrated to 6 mL	Not specified	30 mL of bone marrow concentrated to 2 - 4 mL	35 mL of bone marrow concentrated	Not specified	60 mL of bone marrow concentrated to 3 mL
Viability	Not mentioned	Not mentioned	Not mentioned	Not mentioned	Not mentioned	Not mentioned
Autologous/allogeneic	Autologous	Autologous	Autologous	Autologous	Autologous	Autologous
Concentration method	Concentrated by Smart PReP1 system	Concentrated by IORG-1 system	Harvest1 BMAC system	Regen Global CCR Kit®	Arteriocyte Magellan autologous platelet separation system	Arteriocyte Magellan autologous platelet separation system
Patient phenotype	n = 40, aged 30.2 ± 9.7 years, osteochondral lesions of the talus (OLT)	n = 15, aged 31 ± 7.8 years, OLT	n = 49, osteochondral lesions of the talus (OCL)	n = 94, aged 37.3 ± 14.4 years, ankle osteochondral defects (OCD)	n = 60, aged 36.7 - 38.1 years, OLT	n = 54, OLT
Treating diseases	Osteochondral Lesions of the Talus	Osteochondral Lesions of the Talus	Osteochondral Lesions of the Talus	Ankle Osteochondral Defects	Osteochondral Lesions of the Talus	Osteochondral Lesions of the Talus
Safety	No infection or thrombosis	Not mentioned	1 case of deep vein thrombosis (DVT), 1 case of hip pain	10 cases of arthroscopy, 4 cases of complex regional pain syndrome (CRPS)	No transplantation failure	No serious adverse events

PMID	25662594	27994720	30321966	35203279	34786970	30424945
Source	BMAC (iliac aspiration)	mBMAC vs mACI	BMAC	BMAC	BMAC	BMAC

Cell counts	60 mL of bone marrow concentrated to 6 mL	Not specified	30 mL of bone marrow concentrated to 2 - 4 mL	35 mL of bone marrow concentrated	Not specified	60 mL of bone marrow concentrated to 3 mL
Viability	Not mentioned	Not mentioned	Not mentioned	Not mentioned	Not mentioned	Not mentioned
Autologous/allogeneic	Autologous	Autologous	Autologous	Autologous	Autologous	Autologous
Concentration method	Concentrated by Smart PReP1 system	Concentrated by IORG-1 system	Harvest1 BMAC system	Regen Global CCR Kit®	Arteriocyte Magellan autologous platelet separation system	Arteriocyte Magellan autologous platelet separation system
Patient phenotype	n = 40, aged 30.2 ± 9.7 years, osteochondral lesions of the talus (OLT)	n = 15, aged 31 ± 7.8 years, OLT	n = 49, osteochondral lesions of the talus (OCL)	n = 94, aged 37.3 ± 14.4 years, ankle osteochondral defects (OCD)	n = 60, aged 36.7 - 38.1 years, OLT	n = 54, OLT
Treating diseases	Osteochondral Lesions of the Talus	Osteochondral Lesions of the Talus	Osteochondral Lesions of the Talus	Ankle Osteochondral Defects	Osteochondral Lesions of the Talus	Osteochondral Lesions of the Talus
Safety	No infection or thrombosis	Not mentioned	1 case of deep vein thrombosis (DVT), 1 case of hip pain	10 cases of arthroscopy, 4 cases of complex regional pain syndrome (CRPS)	No transplantation failure	No serious adverse events
